# Application of Low-Cost Me-N-C (Me = Fe or Co) Electrocatalysts Derived from EDTA in Direct Methanol Fuel Cells (DMFCs)

**DOI:** 10.3390/ma11071193

**Published:** 2018-07-12

**Authors:** Carmelo Lo Vecchio, Antonino Salvatore Aricò, Vincenzo Baglio

**Affiliations:** C.N.R., Istituto di Tecnologie Avanzate per l’Energia “Nicola Giordano” (ITAE), Via Salita Santa Lucia sopra Contesse, 5, 98126 Messina, Italy; arico@itae.cnr.it

**Keywords:** non-PGM catalysts, DMFCs, methanol-tolerant catalysts, oxygen reduction reaction

## Abstract

Co-N-C and Fe-N-C electrocatalysts have been prepared by mixing Fe or Co precursors, ethylene diamine tetra acetic acid (EDTA) as a nitrogen source, and an oxidized carbon. These materials were thermally treated at 800 °C or 1000 °C under nitrogen flow to produce four samples, named CoNC8, CoNC10, FeNC8, and FeNC10. They have been physicochemically characterized by X-ray photoelectron spectroscopy (XPS) and transmission electron microscopy (TEM). Direct methanol fuel cell (DMFC) analyses have been carried out to investigate the performance of the nonprecious cathode catalysts, using a low amount of Pt (0.7 mg/cm^2^) at the anode side. It appears that FeNC8 is the best performing low-cost cathode catalyst in terms of higher oxygen reduction reaction activity and methanol tolerance.

## 1. Introduction

Direct methanol fuel cell (DMFC) technology is very promising for portable power source applications [1,2,3,4]. The anode and the cathode of the fuel cell are located at the external side of the solid polymeric electrolytic membrane. The methanol oxidation reaction (MOR) occurs at the anode onto Pt-based catalysts [5,6,7], whereas the oxygen reduction reaction (ORR) occurs at the cathode, also in this case usually based on Pt catalysts [8,9,10,11,12]. A polymeric membrane, generally Nafion^®^ [13,14], is placed between the anode and cathode, allowing the migration of H^+^ produced during methanol oxidation at the anode. One of the main drawbacks of this technology is the use of expensive materials, such as catalysts and membranes. In this regard, non-Pt-group-metal (PGM) catalysts, such as N-doped carbon [15,16,17,18,19] or metal-nitrogen-carbon (Me-N-C) [20,21,22,23,24,25,26], have attracted remarkable interest in the last decades for their promising performance and the great abundance of their precursors in the earth. Another issue to be solved for DMFC technology is the methanol crossover from the anode to the cathode side through the membrane. This effect generates a mixed potential at the cathode that decreases the overall efficiency. The research in this field is addressed to the development of innovative methanol impermeable membranes [27,28,29] or methanol tolerant cathode catalysts [30,31,32,33], the latter being the aim of the present work.

Here, in-house Me-N-C (Me = Fe or Co) cathode catalysts have been synthesized by a low-cost and environmentally friendly nitrogen precursor (ethylene diamine tetra acetic acid, EDTA) and from a high-surface functionalized carbon (Ketjenblack). The investigation was carried out for catalysts treated at different temperatures: catalysts derived from a heat treatment at 800 °C have been labelled as FeNC8 and CoNC8, depending on the chelated metal (Fe or Co), whereas the heat treatment at 1000 °C produced two samples named FeNC10 and CoNC10. The mentioned catalysts deposited at the cathode of a DMFC have been electrochemically characterized to evaluate their performance.

## 2. Materials and Methods

### 2.1. Preparation of Co-N-C- and Fe-N-C-Based Electrocatalysts

**Figure 1:**
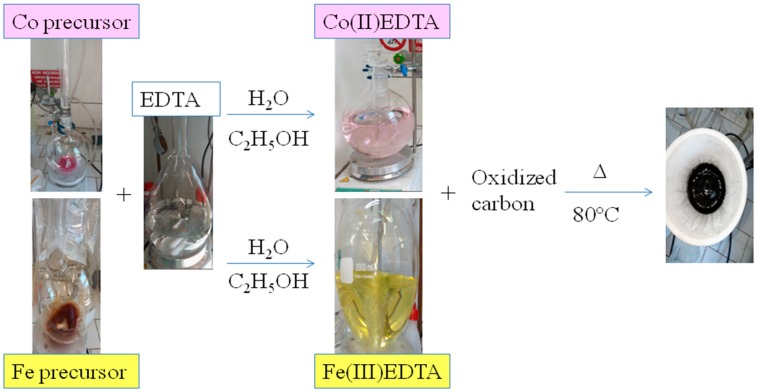
Representation of synthesis procedure for Co-ethylene diamine tetra acetic acid (EDTA) and Fe-EDTA.

The synthesis of the metal-nitrogen-carbon (Me-N-C) compounds was performed by mixing a previously oxidized carbon (Ketjenblack) with Co-chelate or Fe-chelate complexes, as described elsewhere [34] and schematically represented in Figure 1. Briefly, the Co-EDTA and Fe-EDTA complexes were prepared using a similar procedure reported in the literature [35]. EDTA was dissolved in H_2_O/CH_3_CH_2_OH and added dropwise to a Co^2+^ or Fe^3+^ solution. The chelated formation of the pink sol–gel Co-EDTA and soft yellow Fe-EDTA was confirmed by UV–Vis measurements [34]. Hence, a purified and oxidized carbon was mixed into the two sol–gel products and kept under stirring overnight [36,37]. The solvents were evaporated and the impregnated product was dried in an oven under vacuum at 80 °C.

The obtained powder was thermally treated at 800 °C in an inert atmosphere (N_2_) to form CoNC8 or FeNC8. Another portion was thermally treated at 1000 °C in an inert atmosphere (N_2_) to form CoNC10 or FeNC10.

### 2.2. Physicochemical Characterization

Transmission electron microscopy (TEM) analysis was performed on a CM12 microscope (Philips, Eindhoven, The Netherlands). The catalyst powder was first dispersed in isopropyl alcohol; then, some drops of the solution were placed on carbon film-coated Cu grids and successively analyzed by TEM. The surface characteristics of the catalysts were analyzed by X-ray photoelectron spectroscopy (XPS), using a 5800-01 spectrometer (Physical Electronics (PHI), Chanhassen, MN, USA) under the conditions reported in previous paper [34]. Finally, the percentage of nitrogen and carbon in Co-N-C and Fe-N-C catalysts was determined by CHNS analysis, whereas the total metal content was determined by energy dispersive X-ray (EDX) spectroscopy at 25 kV using an XL30 SFEG scanning electron microscope (FEI, Eindhoven, The Netherlands).

### 2.3. Electrochemical Measurements

Regarding the DMFC analysis, the electrodes were prepared using, as backing layers at the anode and cathode, commercial carbon cloths coated with a gas-diffusion layer, HT-ELAT and LT-ELAT (from E-TEK), respectively. The procedure for the preparation of the catalytic ink is reported elsewhere [38]. In order to manufacture the cathodic electrode, FeNC8, FeNC10, CoNC8, and CoNC10 catalysts were dispersed in isopropyl alcohol; successively, a 45 wt % Nafion^®^ ionomer (Ion Power, 5 wt % solution) was added to the solution under sonication. Finally, this ink was deposited by a spray technique onto the LT-ELAT backing layer. A Fe-N-C or Co-N-C loading of 4.0 ± 0.2 mg·cm^−2^ was used at the cathode of all electrodes [22]. In order to manufacture the anodic electrode, an unsupported PtRu catalyst (from Alfa Aesar) was mixed with water and 15 wt % Nafion^®^ ionomer (Ion Power, 5 wt % solution) under sonication. This anodic ink was deposited by a doctor blade technique onto the HT-ELAT backing layer. A Pt + Ru loading of 1 ± 0.1 mg·cm^−2^ was used at the anode in the different membrane electrode assemblies (MEAs). The latter were obtained by hot pressing the electrodes onto both sides of a Nafion^®^ 115 membrane at 130 °C and 20 kgf·cm^−2^ for 1.5 min. The MEAs were investigated in a 5-cm^2^ single-cell hardware connected to a Fuel Cell Technologies Inc. test station. Fully humidified oxygen was fed to the cathode at a flow rate of 100 mL·min^−1^ under atmospheric pressure, whereas 2 mL·min^−1^ methanol solution was fed to the anode at different concentrations (1, 2 M). The performance of each MEA was determined under steady-state conditions at 90 °C.

## 3. Results and Discussion

### 3.1. TEM Analysis

**Figure 2:**
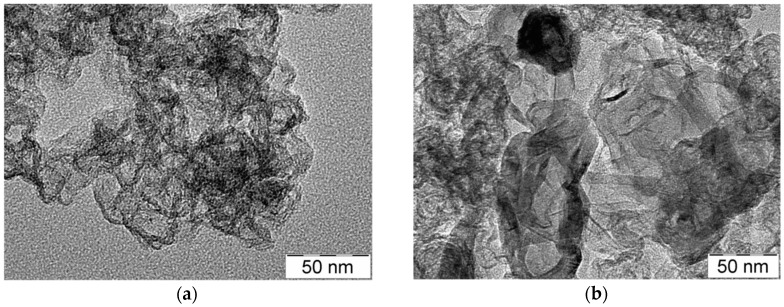

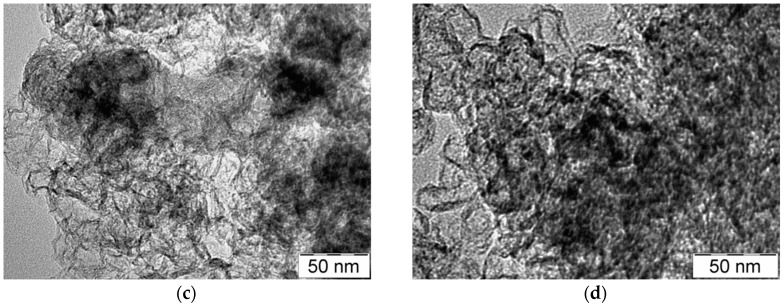
Transmission electron microscopy (TEM) images for the following catalysts: (**a**) CoNC8; (**b**) CoNC10; (**c**) FeNC8; and (**d**) FeNC10.

Figure 2a–d shows TEM images of the Co-N-C or Fe-N-C samples treated at 800 °C or 1000 °C. All the electrocatalysts are characterized by some zones in which the particles are very small (<0.5 nm) and well dispersed, whereas particles agglomeration occurs in other regions. As expected, the higher the heat treatment (1000 °C), the larger the particle size, and this is clearly shown in the image in Figure 2b for CoNC10. However, the other electrocatalysts exhibit a better dispersion and an ordered nanocluster structure, especially CoNC8 (Figure 2a). The formation of empty space in the carbon support is related to the oxidation treatment carried out by using concentrated HNO_3_ (see Materials and Methods section).

### 3.2. X-ray Photoelectron Spectroscopy (XPS) Investigation

**Figure 3:**
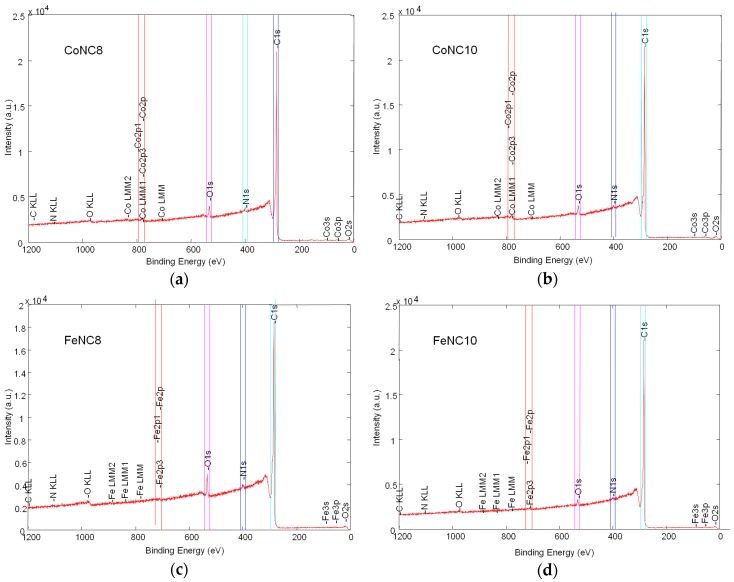
Survey X-ray photoelectron (XPS) spectra for the following catalysts: (**a**) CoNC8; (**b**) CoNC10; (**c**) FeNC8; and (**d**) FeNC10.

XPS data were interpreted using the analyses reported in the literature [15,39,40,41,42,43]. The survey spectra of all the abovementioned electrocatalysts are shown in Figure 3a–d.

The unmarked peak at 315 eV corresponds to the shakeup line of C 1s signal that is the predominant species in such compounds, with an amount ranging from 93.5% to 97.8%. The atomic percentage of O 1s and N 1s is smaller for the catalysts treated at a higher temperature, whereas the metal content derived from XPS analyses is below 0.4% for all the electrocatalysts. From CHNS analysis, the content of nitrogen is 3.3% for CoNC8, 1% for CoNC10, 2.5% for FeNC8, and 1.2% for FeNC10, whereas the atomic percentage of C ranges from 78.1% to 80.4%. From the EDX results, the Co amount is 2.4% for CoNC8 and 1.4% for CoNC10, while Fe metal is present at 2.8% and 2.9% in FeNC8 and FeNC10, respectively.

**Figure 4:**
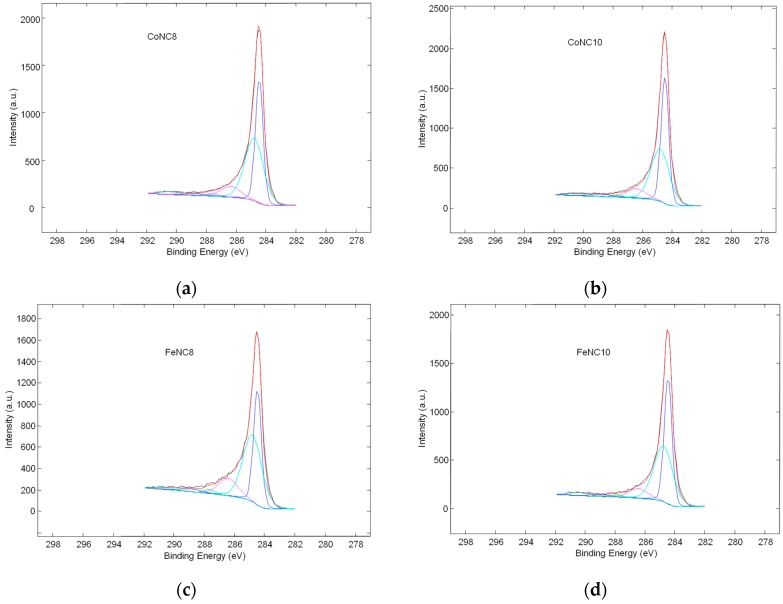
Deconvolution of C 1s high-resolution XPS spectra for the following catalysts: (**a**) CoNC8; (**b**) CoNC10; (**c**) FeNC8; and (**d**) FeNC10.

C 1s deconvoluted spectra of all the thermally treated electrocatalysts are shown in Figure 4a–d.

For Me-N-C electrocatalysts, graphitic carbon occurs at 284.5 (±0.1) eV, C–C, C–(C–N), or carbon oxides bonds at 284.9 (±0.1) eV, C–N_x_ defects at 286.3 (±0.1) eV, C–O–H/C–O–C at 287.5 (±0.1) eV, C=O at 288.7 (±0.1) eV, and COOH at 290.4 (±0.1) eV.

**Figure 5:**
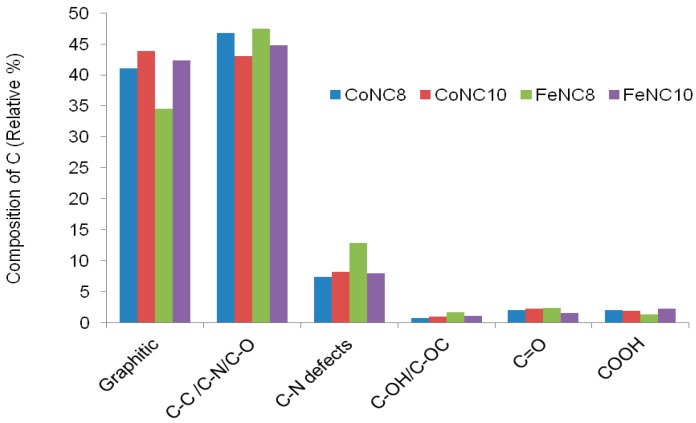
Relative percentage composition of C 1s for CoNC8, CoNC10, FeNC8, and FeNC10 catalysts.

**Figure 6:**
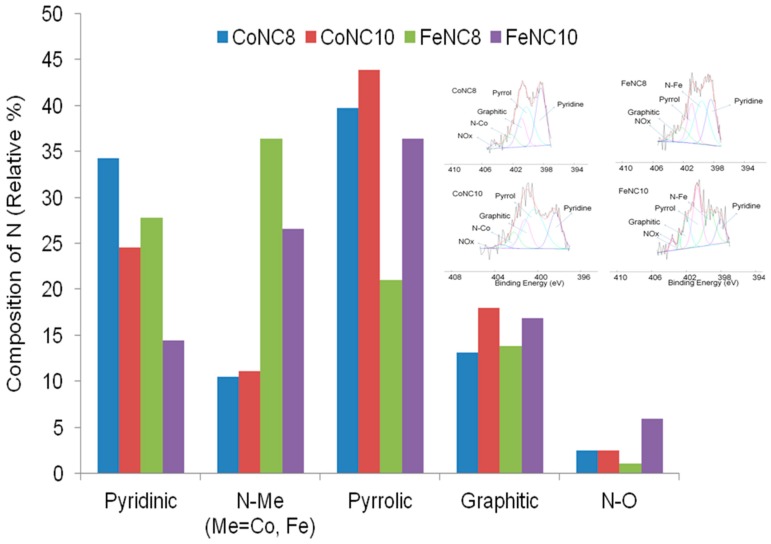
Relative percentage composition and inset of the deconvolution of N 1s XPS signal for CoNC8, CoNC10, FeNC8, and FeNC10 catalysts.

**Figure 7:**
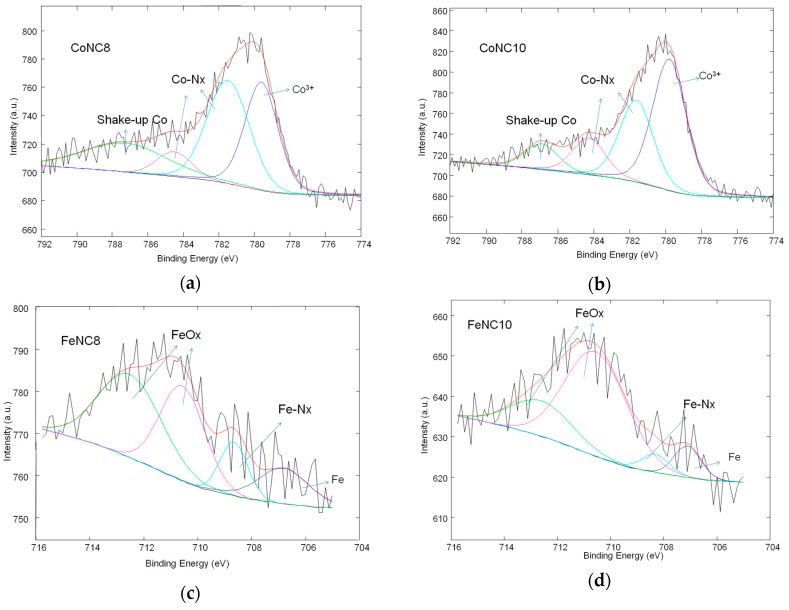
Deconvolution of Co 2p and Fe 2p high-resolution XPS spectra for the following catalysts: (**a**) CoNC8; (**b**) CoNC10; (**c**) FeNC8; and (**d**) FeNC10.

Figure 5 shows the relative amount of the different C species for the various Me-N-C compounds. FeNC8 has a smaller relative amount of graphitic carbon followed by CoNC8 catalyst. Moreover, FeNC8 presents the largest number of C–N_x_ defects, probably due to the higher formation constant of Fe-EDTA compared to Co-EDTA and to a minor temperature of the heat treatment. The deconvolution of XPS spectra related to N 1s signals is shown in Figure 6 (inset), whereas the histograms in Figure 6 are useful to summarize some aspects. Regarding Co-N-C electrocatalysts, pyridinic–N appears at 398.3 (±0.1) eV, pyrrolic–N at 400.2 (±0.1) eV, graphitic–N at 401.1 (±0.1) eV, N_x_–Co at 402.2 (±0.1) eV, and oxidized nitrogen species at a binding energy larger than 403 eV. Regarding Fe-N-C electrocatalysts, pyridinic-N is found at 398.2 (±0.1) eV, N_x_–Fe at 399.5 (±0.1) eV, pyrrolic–N at 400.8 (±0.1) eV, graphitic–N at 402.1 (±0.1) eV, and oxidized nitrogen species at more than 403 eV. It can be seen that FeNC8 exhibits the largest amount of N-Fe interaction and the smallest amount of pyrrolic nitrogen species. Pyridinic sites (known for high ORR activity together with graphitic nitrogen [44,45,46]) have been mainly found in the compounds treated at 800 °C compared to those treated at 1000 °C. Co 2p and Fe 2p deconvoluted spectra for all the abovementioned electrocatalysts are shown in Figure 7a–d. The peak at a binding energy of 779.7 (±0.1) eV is attributed to Co^3+^, the peaks at 781 and 784.4 (±0.1) eV are ascribed to Co–N_x_, and a shakeup peak of elemental Co appears at 786.4 (±0.1) eV. Regarding the FeNC compounds, metallic Fe is characterized by a peak at 706.9 (±0.1) eV, Fe–N_x_ at 708.4 (±0.1) eV, and FeO_x_ between 710 and 713 eV. These results are affected by the notable noise in the signal due to the relatively low metal amount for all the electrocatalysts. However, it can be derived that CoNC8 and FeNC8 present a larger amount of Me–N_x_ and metallic species than CoNC10 and FeNC10. On the other hand, Co and Fe oxides seem to be more relevant in the compounds treated at 1000 °C.

**Figure 8:**
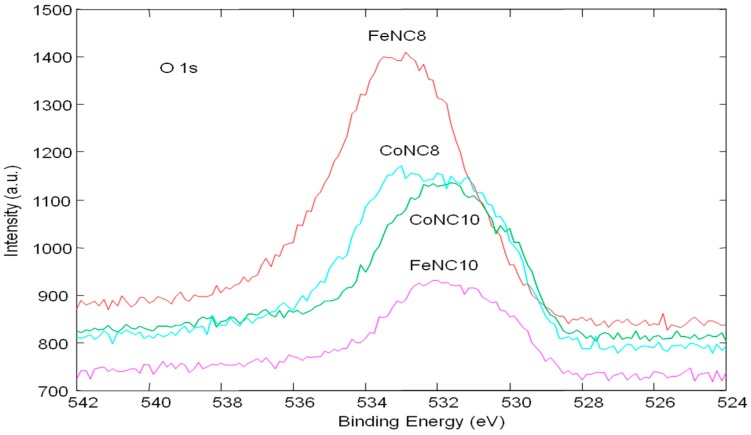
O 1s high resolution XPS spectra for CoNC8, CoNC10, FeNC8, and FeNC10 catalysts.

O 1s high-resolution spectra were reported in Figure 8 after an accurate calibration of C 1s signal. For O 1s, three important contributions have been considered: metal oxides (below 531.5 eV), NO_x_, and organic oxides (above 531 eV), depending on the difference of electronegativity. The metals oxides have a larger difference of electronegativity than organic oxides and NO_x_. Thus, the binding energy of oxygen bound to the metal is lower than that bound to carbon and nitrogen species. O 1s peak for FeNC8 appears to be shifted towards higher binding energy, followed by CoNC8. It means that the samples heat treated at a lower temperature are characterized by a higher amount of organic oxides and NO_x_ interactions, whereas a significant contribution of metal oxide is observed for FeNC10 and CoNC10.

### 3.3. Direct Methanol Fuel Cell (DMFC) Characterization

**Figure 9:**
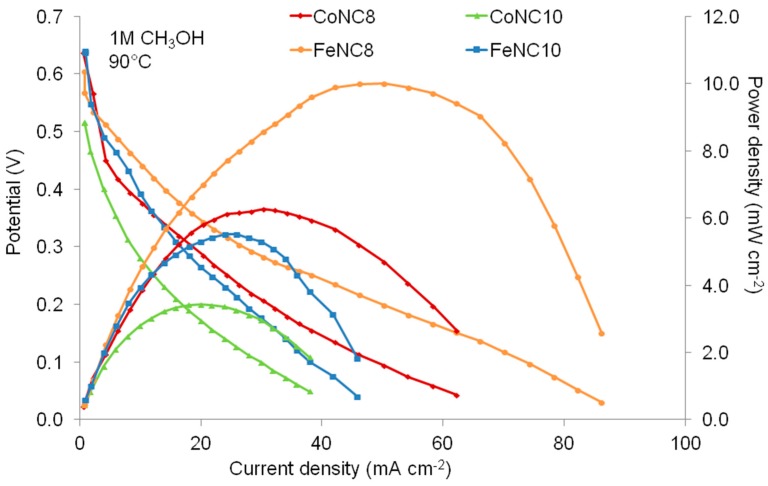
Polarization and power density curves obtained for CoNC8, CoNC10, FeNC8, and FeNC10 at the cathode of the direct methanol fuel cell (DMFC) by using a 1 M methanol concentration at 90 °C.

DMFC polarization and power density curves for the various membrane electrode assemblies (MEAs) equipped with the synthesized Me-N-C catalysts, carried out at 90 °C in the presence of 1 M methanol solution at the anode, are shown in Figure 9. Under these operating conditions, but at lower temperatures too (not shown), the best performing MEA is the one based on the FeNC8 cathode, with a power density almost twice as high as that with the best performing cathode treated at a higher temperature (FeNC10). The same difference, in terms of maximum power density, is obtained by comparing the CoNC8 with CoNC10 cathode-based MEAs.

These results have been analyzed in terms of the physicochemical properties observed by TEM and XPS (C 1s and N 1s composition) measurements. It appears that the worst electrocatalyst (CoNC10) is also characterized by the worst metal-particle distribution on the support and, thus, the largest amount of agglomeration. Furthermore, this catalyst presents the largest content of pyrrolic nitrogen. In fact, it is believed that these species cause a performance loss due to the partial oxygen reduction and consequent formation of H_2_O_2_ instead of H_2_O [30]. Also, CoNC8 and FeNC10 show a large amount of pyrrolic nitrogen species, which explains the low performance of the MEA based on these catalysts.

**Figure 10:**
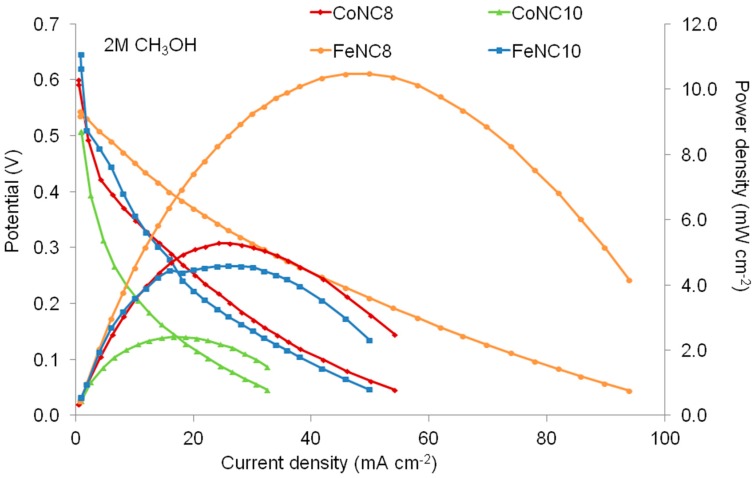
Polarization and power density curves obtained for CoNC8, CoNC10, FeNC8, and FeNC10 at the cathode of the DMFC by using a 2 M methanol concentration at 90 °C.

Figure 10 shows the polarization and power density curves by feeding 2 M methanol solution at the anode side. The best MEA based on the FeNC8 cathode achieves a maximum power density of 10.5 mW·cm^−2^, maintaining a performance similar to that obtained with 1 M methanol solution. On the other hand, the performance slightly decreases for the other MEAs, passing from 1 to 2 M methanol solution. The performance slightly increases from 1 M to 2 M methanol for FeNC8, proving a greater tolerance to the poisoning caused by the alcoholic fuel at the cathode (due to the methanol crossover through the membrane) compared to the other cathode based on Me-N-C catalysts. Fe-N-C nanoclusters (treated at 800 °C) do not present the best particle distribution on the support, as verified by TEM, but they are characterized by the largest percentage of C-N defects. Another relevant feature of the best catalyst is the most positive ratio between pyridinic and pyrrolic nitrogen and the highest N-Fe interaction. The latter is supposed to be the main active site for the oxygen reduction reaction (ORR), which leads to the enhancement of the performance in a DMFC. For the catalyst based on cobalt, the percentage of N-Co groups is quite low; this explains the lower activity of this catalyst for the ORR and, accordingly, a lower performance in the DMFC. These results are preliminary and require further improvement to obtain a large-scale commercialization of cost-effective DMFCs.

The performance of a benchmark electrocatalyst (Pt/C 1 mg Pt cm^−2^ at the cathode) is eightfold higher than that obtained with these novel electrocatalysts, as reported in the literature [47]. On the other hand, Fe-N-C catalysts represent a considerable option for DMFC cathodes due to the good results obtained in some previous works [30,48,49], mainly in terms of activity and methanol tolerance. Here, a facile preparation of nonprecious catalysts based on a template-free procedure has been carried out. The obtained catalysts have been investigated in a DMFC under reliable conditions (low-Pt loading at the anode), giving rise to a maximum power density comparable with recently published papers [44,50,51]. Certainly, there are other works where higher power densities are reported [30,49]; however, these deal with catalysts prepared by using complex preparation procedures (use of templates and their removal with aggressive reactants), high-cost precursors, etc. Instead, in this work, the metals are derived from metal nitrates, the nitrogen precursor is a low-cost and easily available bio-compound (EDTA), and a template-free preparation procedure is involved. From the present results, it is clear that an optimization of the synthesis procedure, which could improve the poor solubility of the iron precursors in H_2_O/CH_3_CH_2_OH and lead to a more organized nanocluster structure, is necessary. Furthermore, a functionalization of the nitrogen precursor should be beneficial for obtaining more active oxygen reduction catalysts based on non-noble metals and earthly abundant compounds.

## 4. Conclusions

Me-N-C (Me = Co or Fe) cathode catalysts were prepared by chelating the metal with a biological nitrogen precursor (EDTA), followed by deposition on a high surface carbon support. In a successive step, the composite materials were thermally treated at 800 °C (CoNC8 and FeNC8) or 1000 °C (CoNC10 and FeNC10) in a nitrogen atmosphere. The DMFC performance was correlated with the physicochemical properties obtained by TEM and XPS analyses. It appears that FeNC8 is the best performing electrocatalyst, showing the largest percentage of C-N defects, pyridinic/pyrrolic ratio, and N-Fe interaction.
